# Industry 4.0 enabled calorimetry and heat transfer for renewable energy systems

**DOI:** 10.1016/j.isci.2025.112994

**Published:** 2025-06-25

**Authors:** Emmanuel O. Atofarati, Christopher C. Enweremadu

**Affiliations:** 1 Department of Mechanical, Bioresources and Biomedical Engineering, University of South Africa, Science Campus, Florida, Johannesburg 1710, South Africa

**Keywords:** Heat transfer, Energy engineering, Energy sustainability

## Abstract

The integration of the fourth industrial revolution technologies, including artificial intelligence (AI), machine learning (ML), the Internet of Things (IOT), digital twins, and blockchain, is advancing calorimetry and heat transfer in renewable energy systems. This review examines how these technologies improve thermal efficiency, enable real-time system monitoring, and support predictive maintenance across solar, wind, geothermal, and bioenergy applications. AI-driven models are discussed for optimizing complex heat transfer behaviors, while IoT frameworks facilitate continuous calorimetric data acquisition. Digital twins support virtual simulations, and blockchain ensures data security. A comprehensive evaluation of recent research identifies key challenges such as computational demands, data security, and policy gaps. The article proposes future directions such as developing hybrid AI-physics models, enhancing explainable AI, conducting long-term performance validation, and standardization frameworks to enable the reliable deployment of smart thermal management systems for renewable energy.

## Graphical abstract

**Figure:**
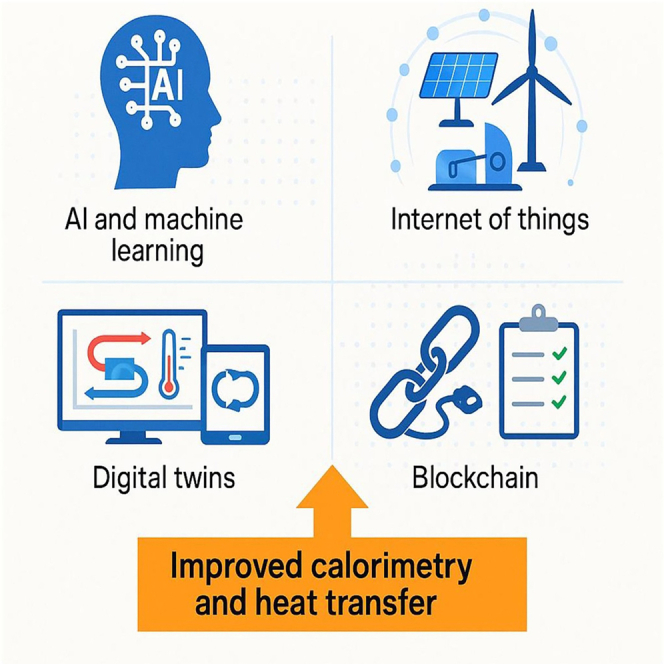

## Introduction

The global shift toward sustainable energy has heightened the need to enhance the performance, reliability, and monitoring of renewable energy systems. Central to this effort is the accurate assessment and optimization of heat transfer and calorimetry processes, which are crucial for energy conversion, thermal storage, and efficiency management across various platforms.^1^ Traditional methods often fall short due to issues such as environmental losses, delayed processing, and human error.^2^^,^^3^ To address these limitations, the Fourth Industrial Revolution (4IR) introduces transformative digital technologies, such as artificial intelligence (AI), machine learning (ML), the Internet of Things (IoT), digital twins, and blockchain, which are redefining how thermal data are captured, processed, and utilized.^4^^,^^5^

The integration of 4IR technologies into calorimetry and heat transfer is particularly relevant as the renewable energy sector faces critical challenges, including energy intermittency, inefficiencies in thermal energy storage, and real-time system monitoring constraints.^6^^,^^7^ AI-based models have demonstrated superior predictive capabilities for optimizing heat transfer efficiency, with studies reporting R^2^ values for the analysis of test data exceeding 0.99 in solar-assisted thermal systems.^8^ IoT-enabled sensors facilitate real-time data acquisition, enhancing system adaptability and reducing operational losses.^9^ Furthermore, digital twins allow for real-time simulations and predictive maintenance, while blockchain enhances data security and transparency in energy transactions.^10^^,^^11^ These technologies collectively improve energy yield and minimize losses in renewable energy systems such as solar PV/thermal,^12^ wind turbines,^13^ geothermal reservoirs,^14^ and bioenergy units.^15^

4IR has significantly influenced the solar photovoltaic (PV) sector, leading to advancements in efficiency, reliability, and management through the integration of technologies such as artificial intelligence (AI), the Internet of Things (IoT), and blockchain. Ahan et al.^16^ developed an end-to-end fault detection system utilizing electroluminescence (EL) imaging to identify and localize faults within solar panels. Their approach employs a hybrid architecture combining multiple convolutional neural network (CNN) models, achieving a 95% cell-level fault prediction accuracy, thereby enhancing manufacturing efficiency and reducing maintenance costs. To address challenges in maximizing power output under varying environmental conditions, Masry et al.^17^ introduced a hybrid maximum power point tracking (MPPT) technique that integrates artificial neural networks (ANN), variable step perturb and observe (VSP&O), and fuzzy logic controllers (FLC). This method demonstrated rapid tracking speeds (less than 0.1 s) and high efficiency (over 99.65%) in extracting maximum power from partially shaded PV systems.

In the realm of energy management, Li et al.^18^ proposed a secure and optimal energy trading framework among AC, DC, and hybrid microgrids using blockchain technology. They enhanced blockchain security by adding a security layer and employed a whale optimization algorithm (WOA) within a digital twin model to find optimal solutions, effectively managing uncertainties in PV and wind turbine outputs. Furthermore, Rao et al.^19^ developed an IoT-based intelligent energy monitoring system for solar PV power generation. This system collects real-time data on voltage, current, temperature, and irradiance, facilitating continuous monitoring and analysis to predict future power generation and optimize maintenance, thereby improving the reliability and efficiency of PV systems. Collectively, these studies underscore the pivotal role of 4IR technologies in advancing solar PV systems, leading to improved fault detection, power optimization, secure energy management, and intelligent monitoring.

Also, 4IR technologies are also being harnessed to enhance the efficiency and reliability of wind power systems. Ding et al.^20^ developed an AI-based system using support vector machine (SVM) algorithms coupled with image-based diagnostics, enabling remote real-time monitoring of wind power equipment. This system employs pan-tilt cameras to capture real-time images of equipment, which are then transmitted to a remote monitoring center. Utilizing variational mode decomposition to extract abnormal state features from these images, the system inputs this data into a support vector machine to achieve real-time anomaly recognition. Experimental results demonstrated the IOT system’s capability to accurately detect anomalies in wind turbines, offering a more efficient alternative to traditional manual inspections.

In another study, Harrison-Atlas et al.^21^ explored the application of AI in optimizing wind plant layouts through wake steering, a control strategy that directs turbine wakes to enhance overall plant energy production. By integrating AI models trained on engineering wind flow simulations, the researchers evaluated over 6,800 potential onshore wind sites across the United States. Their findings suggest that co-optimizing plant layouts with wake steering can reduce land requirements by an average of 18% per plant, with site-specific benefits ranging from 2% to 34%. Additionally, wake steering is predicted to increase power production during high-value periods, potentially boosting annual revenue by up to US$3.7 million for individual plants. This study underscores the geographic variability in the economic and siting benefits of wake steering, highlighting the importance of considering local factors in wind plant optimization.

Building upon the advancements in AI within renewable energy sectors, recent studies have explored its application in geothermal energy. Moraga et al.^22^ introduced a methodology that integrates remote sensing, machine learning, and AI to assess geothermal potential by analyzing indicators such as mineral markers, surface temperature, faults, and deformation. Their approach demonstrated high accuracy levels between 92% and 95% in predicting geothermal sites, offering a valuable tool for early-stage exploration.

Similarly, Buster et al.^23^ developed the Geothermal Operational Optimization with Machine Learning (GOOML) framework, which combines data-driven and thermodynamic models to optimize geothermal power plant operations. By creating digital twins of geothermal systems, GOOML enables the enhanced exploration of operational spaces, leading to improved efficiency and sustainability in geothermal energy production. These studies highlight the transformative potential of AI in enhancing both the exploration and operational phases of geothermal energy projects, contributing to more efficient and effective utilization of this renewable resource.

Basically, traditional calorimetric methods often suffer from inaccuracies due to erroneous human inspection, inaccurate record keeping, unforeseen maintenance requirements, environmental heat loss, sensor limitations, and delayed data processing, among others.^2^^,^^3^ AI-driven predictive analytics and IoT-based thermal monitoring offer potential solutions to these inefficiencies by eliminating unnecessary experimentation cycles, optimizing system performance, and reducing energy losses.^24^^,^^25^^,^^26^ Moreover, the renewable energy sector must meet stringent environmental regulations, reduce carbon emissions, and improve cost-efficiency. The integration of 4IR-enabled tools can enhance heat transfer measurements, optimize energy utilization, and facilitate predictive maintenance, thereby driving greater sustainability.^6^

Comprehensive evaluations of how AI, IoT, and blockchain synergistically enhance renewable energy thermal management are scarce. Moreover, most AI-driven thermal models are validated on laboratory-scale datasets, lacking long-term field verification. Although AI-based thermal performance optimization is well-studied, comparative analyses of machine learning models and their predictive accuracies in calorimetric applications remain limited. The integration of IoT-enabled real-time monitoring with digital twins for predictive heat transfer modeling is still emerging, with few empirical validations. While solar, wind, geothermal, and bioenergy systems stand to benefit from these advancements, the impact of 4IR technologies on reducing energy losses and scaling large renewable infrastructures remains underexplored.

This review explores the state-of-the-art applications of 4IR technologies in calorimetry and heat transfer, focusing on their role in improving efficiency, reliability, and scalability within renewable energy systems. While existing literature has examined individual technologies such as AI or IoT in isolation, comprehensive assessments of their integrated applications in thermal management remain limited. Prior reviews often overlook comparative analyses of machine learning models for calorimetric prediction, lack real-world validations for digital twin implementations, and rarely address the synergistic use of blockchain for data security in thermal systems. This review aims to bridge these gaps by conducting a critical thematic synthesis of literature published between 2019 and 2025, focusing on applications of AI, IoT, and blockchain in thermal energy optimization across solar, wind, geothermal, and bioenergy systems. The objective is to identify current capabilities, unresolved challenges, and future research opportunities that can accelerate the development of intelligent, secure, and scalable heat transfer solutions for renewable energy infrastructures.

## Literature search and data collection method

A systematic literature review was conducted to gather relevant studies on the integration of Industry 4.0 (4IR) technologies such as artificial intelligence (AI), machine learning (ML), the Internet of Things (IoT), digital twins, and blockchain into calorimetry and heat transfer applications in renewable energy systems.

The search was performed using Scopus, Web of Science, and IEEE Xplore databases, covering the publication period from January 2019 to March 2025. The following inclusion criteria were applied.

(1) Peer-reviewed journal articles and high-impact conference proceedings
(2) English language publications
(3) Studies explicitly addressing 4IR applications in thermal systems, calorimetry, or renewable energy
(4) Empirical research, technical reviews, or implementation case studies

Search strings were formulated using combinations of keywords such as.

(1) (“calorimetry” OR “heat transfer”) AND (“artificial intelligence” OR “machine learning”)
(2) (“IoT” OR “Internet of Things”) AND (“thermal systems” OR “energy efficiency”)
(3) (“digital twin” OR “cyber-physical systems”) AND (“renewable energy”)
(4) (“blockchain” OR “data security”) AND (“thermal energy” OR “smart grid”)

**Figure 1:**
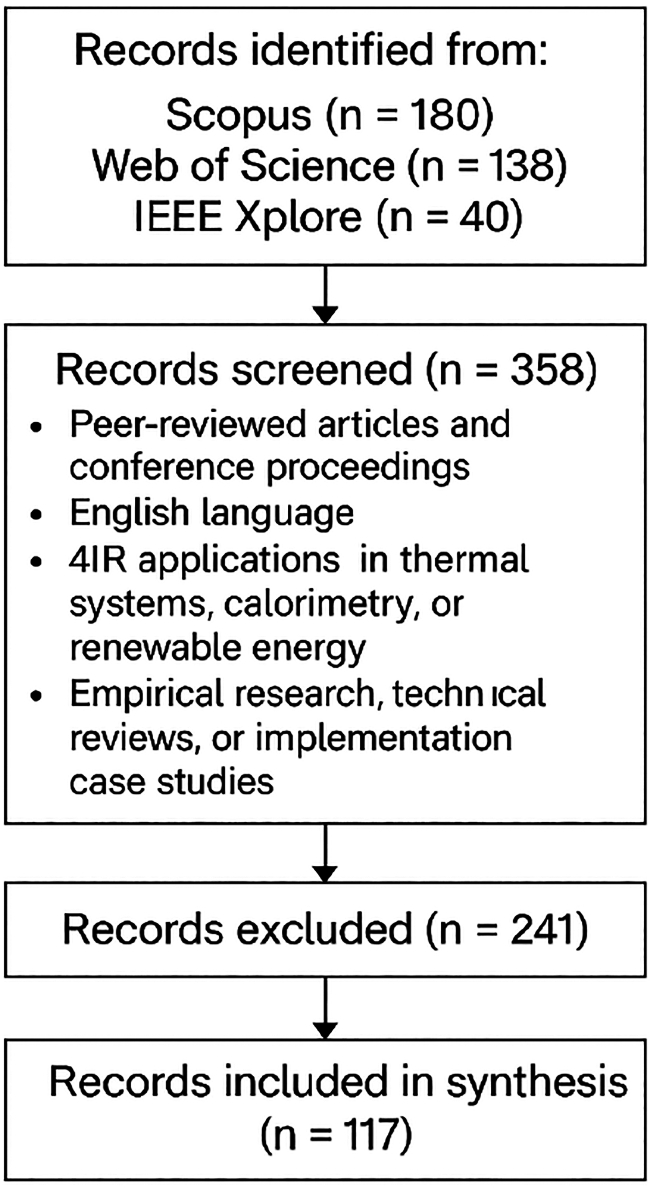
Prisma Chart for Industry 4.0-Enabled Calorimetry and Heat Transfer

A total of about 358 articles were initially retrieved. After title/abstract screening and full-text review based on relevance, duplication, and quality criteria, 117 articles were selected for detailed thematic analysis. Data were extracted and categorized according to technology type, application domain (solar, wind, geothermal, bioenergy), methodological approach, and reported outcomes. The results of this synthesis form the evidence base for the comparative analyses and case studies presented in Sections Artificial Intelligence (AI) and Machine Learning (ML) in Calorimetry and Heat Transfer, Internet of Things (IoT) for enhanced monitoring and automation, big data analytics and cloud computing for scalable heat transfer optimization, Cyber-Physical Systems and digital twin technology, and blockchain for data security. Figure 1 shows the Prisma Chart for the data search exclusion and the final document screened for this analysis.

## Fourth Industrial Revolution technologies transforming calorimetry and heat transfer

The application of Fourth Industrial Revolution (4IR) technologies in calorimetry and heat transfer is reshaping the renewable energy landscape by improving measurement accuracy, real-time monitoring, and system optimization. Traditional thermal calorimetric techniques, such as Differential Scanning Calorimetry (DSC), Bomb Calorimetry, and Flow Calorimetry, often face challenges including delayed data acquisition, environmental heat losses, and inaccuracies in thermal modeling. These limitations can affect the precision and reliability of measurements in various applications. 4IR-driven innovations offer enhanced solutions for predictive modeling, performance optimization, and real-time system diagnostics. This section discusses key advancements in AI/ML, IoT, and big data analytics in the context of calorimetry and heat transfer within renewable energy systems.

### Artificial intelligence and machine learning in calorimetry and heat transfer

AI and ML have significantly improved the predictive accuracy and automation of calorimetric and heat transfer systems.^27^ These technologies analyze vast datasets, identify patterns, and develop models that optimize thermal performance, wind turbine design, PV cell systems, among others, as summarized in Table 1. Also, AI-driven algorithms enhance decision-making in energy conversion, anomaly detection, and thermal system optimization, leading to improved efficiency and reliability.

**Table 1:** Recent advances in AI and machine learning for calorimetry and thermal systems

Author & Year	AI/ML Method	System/Application Type	Purpose	Significance of AI or ML	Main Outcome	Additional Notes
Said et al.^8^ (2022)	MLP-ANN	Solar-assisted shell and tube heat exchanger (STHX)	Prognostic modeling	High prediction accuracy of test data (R > 0.99)	Enhances heat transfer efficiency (31.08%)	Reduces cost & CO_2_ emissions.
Wang et al.^28^ (2024)	Swin-Unet, FCN8, U-Net	Fire calorimetry	Quantify fire power	Low Heat Release Rate error (<20%)	Enhanced fire monitoring	Useful for digital twin systems.
Ullah et al.^26^ (2023)	ANN, Regression	Nanofluid systems	Predict & optimize nanofluid properties	Enhances CFD accuracy	ML predicts nanofluid properties effectively	Overcomes traditional model limits.
He et al.^29^ (2021)	ANN, SVM, GA, Fuzzy Logic, …	TES systems	Predict, optimize, control TES	Improves TES efficiency & renewables use	Accurate predictions; operational optimization	Lacks comprehensive TES data.
Anooj et al.^30^ (2022)	RNN	PCM-based systems	Predict PCM liquid fraction	Enables real-time PCM monitoring	High test correlation (R = 0.99, RMSE<0.015)	Generalizes well with low computational cost.
Rehman et al.^31^ (2023)	DNN	PCM-porous heat sinks	Predict thermal performance	Accurate for diverse conditions (R^2^ = 0.99, for test data)	MAE = 0.0438; XAI validates key inputs	Explores PCM, foam material effects.
Rathod et al.^32^ (2023)	RFR, DT, Poly Reg, …	Alkanes/refrigerants	Predict thermophysical properties	High %AARD & MAE accuracy	RFR excels across variables	Correlations aid molecular design.
Gao et al.^33^ (2022)	KNN, LASSO, Poly Reg, …	Carbon-based PCMs	Predict PCM properties	High R^2^ (≥0.98), MSE (10^−9^) for test data.	Insights for nano-enhanced PCM design	100 thermal cycles analyzed.
Aldaghi et al.^34^ (2022)	RL-GMDH, RBF, …	Hybrid heat sink	Predict PCB temperature	Accurate predictions (30s runtime)	Enhanced cooling via hybrid methods	Combines deep learning and nanofluids.
Bhattad & Priyadarsan^35^ (2024)	Symbolic Regression	Alumina hybrid nanofluids	Predict thermal conductivity	High accuracy (3.1% deviation)	Validated with experimental data	Explores ternary nanofluids.
Vempally & Dhanarathinam^36^ (2023)	DTR, k-NN, XGBR	SCPCMs for latent heat	Predict heat flow, SHC	Reliable for PCM selection	Accurate for heat flow, SHC	Reduces design cost/time.
Farah et al.^37^ (2020)	RF, GBM, MLP	Milk adulteration detection	Detect adulteration via DSC & ML	100% detection accuracy	DSC identifies adulteration peaks	Effective quality monitoring tool.
Said et al.^38^ (2022)	XGBoost, GPR, SVM	Water-based hybrid nanofluids	Predict SHC	High accuracy (MAPE = 0.01%)	Cost-effective experimental alternative	SHC optimization for solar use.
Olimat et al.^39^ (2022)	ANN	Timber combustibility	Predict burning rates	High R^2^ (0.99292) for the test data	Improves fire safety standards	Supports building codes development.
Sezer et al.^40^ (2024)	ANN, LWR, GBM	Flow boiling systems	Predict HTC, ΔP	High HTC/ΔP accuracy (82.4–88.9%)	LWR excels with limited data	Validated through extrapolation tests.
Bifulco et al.^41^ (2023)	ANN	Mg(OH)2-Epoxy composites	Predict fire capacity	Low error (MAE = 145.6)	Aids fire-resistant material design	Cost-effective method for composites.
Kanti et al.^42^ (2024)	DNN, XGBoost, SHAP	Hybrid nanofluids	Predict/interpret thermophysical props.	Accurate (XGBoost R^2^ = 0.9122)	SHAP highlights key influences	Optimizes nanofluid design.
Atofarati et al.^24^ (2024)	RF, GB, SVM, LR	Nanofluid cooling	Predict thermal performance	RF & GB are accurate for key factors	Boosts heat dissipation	Flow Reynold is the most significant factor.
Amer et al.^43^ (2021)	ML, Transfer Learning	Thermographic CUI detection	Detect pipeline corrosion	Improves detection (85–90%)	Real-time, non-contact inspections	Reduces downtime, enhances safety.
Kumar et al.^44^ (2024)	Ada-Boost, RF	Nanofluid solar absorption	Optimize RM nanofluid properties	36.9% conductivity boost	Eco-friendly solar applications	AdaBoost outperforms RF.
Mishra et al.^45^ (2024)	ANN, RF, SVR	Coal mines	Predict heating risks	Accurate for heating potential	ANN achieves R^2^ = 0.85	Supports safer mining.
Naser^46^ (2021)	DL, RF, SVM	Fire engineering	Tackle fire challenges	Solves complex FEA problems	Effective for multi-dimensional issues	Collaboration/database needs noted.
Adelekan et al.^47^ (2022)	FNN, RBF, ANFIS	HVAC systems	Predict/optimize RHVAC	Gradient issues noted	Effective with proper training	New activation functions needed.
Bashirgonbadi et al.^48^ (2024)	Partial Least Squares (PLS)	PE/PP blends	Predict composition	Low error (1.0 wt %)	Fast, accurate recycled plastic method	Quantifications of sub-category polymers.

#### Artificial intelligence-powered predictive modeling for heat transfer optimization

**Figure 2:**
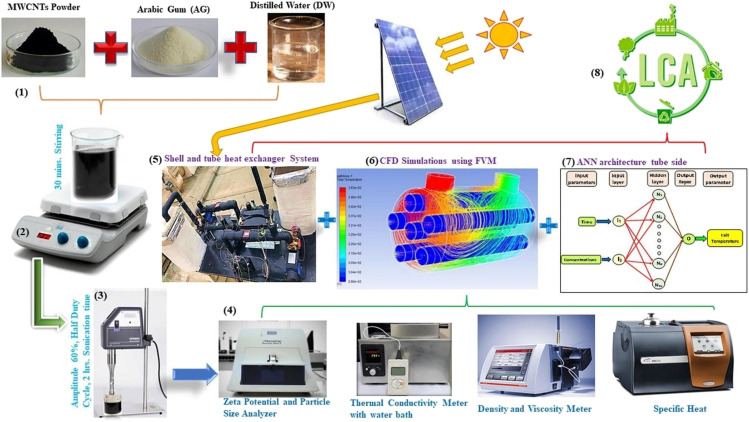
Schematic diagram of a prognostic model predicting the thermal performance of nanofluids in a solar-aided shell and tube heat exchanger over time Reproduced with permission from Elsevier in Said et al.^8^ published by Elsevier.

Recent research demonstrates how AI/ML models outperform conventional analytical methods in predicting thermophysical properties, optimizing energy utilization, and improving the accuracy of heat transfer simulations. A Multilayer Perceptron Artificial Neural Network (MLP-ANN) used to model the thermal performance of a solar-assisted shell-and-tube heat exchanger (STHX) using MWCNT/water nanofluids was investigated by Said et al.^8^ Figure 2 shows the schematics of the methodological approach used in their study. Their model achieved exceptionally high predictive accuracy, with R-values of 0.998 and 0.994 for tube-side and shell-side models, respectively. The AI-based approach improved heat transfer efficiency by 31.08%, demonstrating its potential for optimizing renewable energy applications.

**Figure 3:**
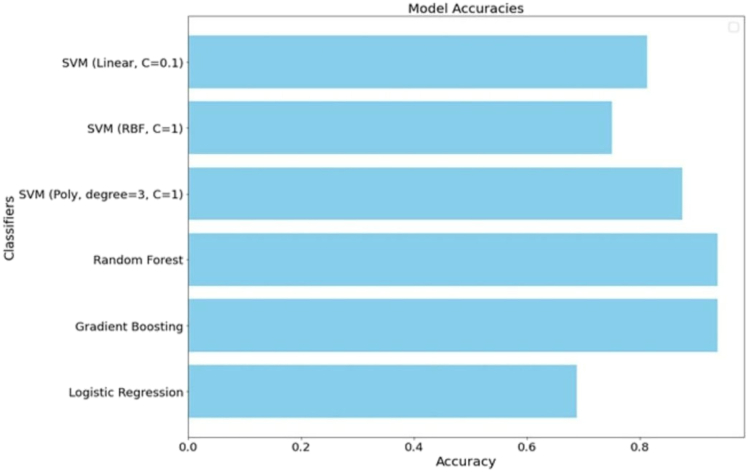
Comparison of different machine learning models for the classification of the factors influencing the Heat transfer Performance in a Jet impingement Cooling system Reproduced from Atofarati et al.^24^ under the CC-BY License, published by Elsevier.

Similarly, studies by Ullah et al.^26^ and Rathod et al.^32^ showed that ML models, including Random Forest Regression and Artificial Neural Networks (ANN), accurately predict thermophysical properties of nanofluids and refrigerants, respectively, surpassing traditional empirical models. Further research by Atofarati et al.,^24^ Gao et al.,^33^ and Bhattad & Priyadarsan^35^ demonstrates the applicability of regression models and AI tools in predicting the properties of nano-enhanced heat transfer performance, phase change materials (PCM), and hybrid nanofluids. Their findings underscore the role of AI/ML in reducing experimental costs and expanding material applications in thermal energy management. Figure 3 provides a visual representation of the predictive accuracy of different AI models in classifying the factors that influence the thermal enhancement in a jet impingement cooling.

These studies highlight the role of AI/ML in enhancing the design and operational efficiency of heat transfer systems. However, despite these advancements, current research lacks comprehensive comparative studies on the performance of different AI models across diverse heat transfer applications. Future studies should explore hybrid AI-physics models that integrate fundamental thermodynamic principles with ML-driven optimizations to further improve prediction accuracy and computational efficiency.

#### Case studies on artificial intelligence and machine learning applications in calorimetry and thermal systems

##### Understandable Deep Learning for Image-Determined Flame Calorimetry

**Figure 4:**
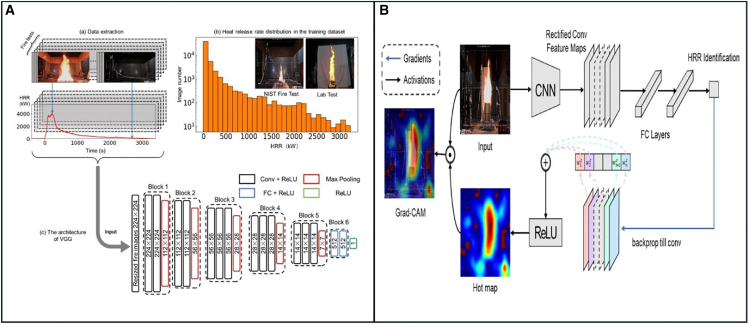
AI-assisted flame calorimetry and fire detection architecture Flame Calorimetry using AI (i) (A) the data extraction process, (B) the distribution of heat release rate (HRR) in the dataset, and (C) the modified VGG model architecture for fire detection (ii) Visualization of the fire calorimetry model using Grad-CAM. Adapted with permission from Wang et al.^28^ published by Springer Nature.

The accurate quantification of fire power is critical for fire detection, monitoring, and control, yet traditional measurement methods are often intrusive and unsuitable for dynamic scenarios. Wang et al.^28^ introduced an AI-driven approach that utilizes explainable deep learning to estimate fire power from flame imagery. A diverse flame image database was constructed, including original RGB, background-free RGB, grayscale, and binary images. A pre-trained fire segmentation model enhanced the accuracy of flame analysis under varying conditions, as shown in Figure 4.

Key findings revealed that flame area was the dominant factor in fire power estimation, with improved segmentation accuracy reducing estimation errors to below 20%. Additionally, Gradient-weighted Class Activation Mapping (Grad-CAM) provided insights into the AI model’s decision-making process. This research highlights the potential of integrating explainable AI for real-time fire monitoring and smart firefighting, offering both accuracy and transparency in thermal system management.

##### Deep Learning-Enhanced e-Skin for Thermoregulation

**Figure 5:**
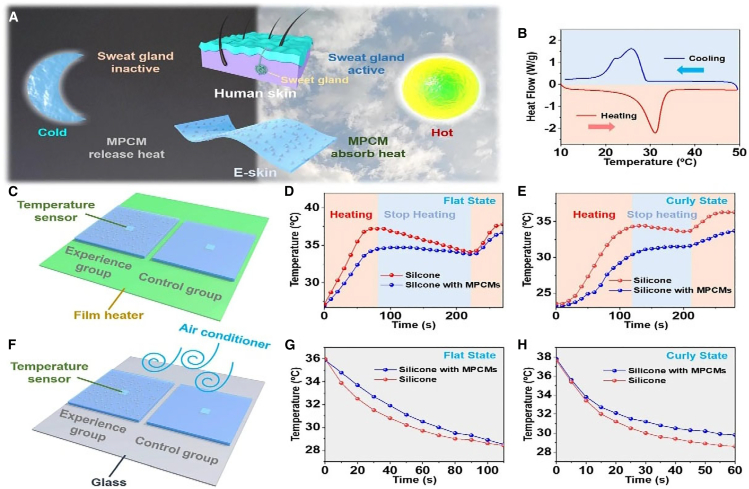
Dynamic thermoregulation behavior of ME-skin under thermal stimuli (A) Schematic inspired by human sweat glands, (B) DSC curve, (C) temperature setup with ME-skin and control layers, (D) temperature variation over time on a flat surface, (E) response of curly ME-skin to heating, (F) temperature changes under air conditioning, (G) cooling behavior at 16°C, and (H) time-temperature curve comparison. Adapted from Xiang et al^.^^49^ under the CC-BY License, Published by Nature.

Advancements in AI-driven thermal systems have enabled the development of multifunctional wearable technologies, such as deep learning-enabled electronic skin (e-skin) with handwriting recognition and thermoregulating capabilities was detailed in the study by Xiang et al.^49^ This system integrates sensors and deep learning algorithms to accurately capture handwriting motions while dynamically adjusting thermal properties for user comfort. The AI model exhibited high accuracy in recognizing individual handwriting styles, ensuring precise data interpretation as presented in Figure 5. Additionally, its adaptive thermal management made it suitable for prolonged use across varying environmental conditions. The seamless combination of handwriting recognition and thermoregulation underscores the growing potential of AI-driven wearable electronics, with applications extending to healthcare, human-machine interaction, and personalized thermal comfort solutions.^49^

##### Smart Autonomous IoT Fire Alert System

To address challenges in conventional fire warning systems, Ding et al.^11^ developed an intelligent, self-powered fire warning system leveraging flexible thermoelectric composite films composed of single-walled carbon nanotubes (SWCNTs) and titanium carbide (Ti_3_C_2_). These films demonstrated high thermoelectric performance, excellent stability at elevated temperatures, and flame retardancy. The thermoelectric devices (TEDs) detected temperature variations and generated voltage signals to trigger fire warnings, with adjustable sensitivity ranging from 1 mV to 10 mV. The system achieved an ultrafast response time of 0.1 s at the lowest threshold, ensuring rapid fire detection. Moreover, IoT integration facilitated remote monitoring via Bluetooth-enabled communication. This innovation enhances fire safety by combining energy efficiency, real-time data accessibility, and adaptability for diverse environments, ranging from residential buildings to industrial facilities.^11^

##### Forecasting impulsive heating in underground coal mines

Spontaneous heating in deep coal mines presents significant safety risks, traditionally assessed through extensive laboratory testing. Applied AI and ML techniques were used by Mishra et al.^45^ to predict spontaneous heating propensity in the Jharia Coalfield (JCF), using data from 55 coal samples collected from depths of 64–675 meters. Principal Component Analysis (PCA) identified key parameters such as moisture content, volatile matter, specific surface area, and depth as critical to spontaneous heating. Predictive models employing artificial neural networks (ANN), random forests (RF), and support vector regression (SVR) were developed, with ANN demonstrating the highest accuracy (R^2^ = 0.80 for crossing point temperature and 0.85 for ignition temperature).

The study revealed that lower moisture content (0.45–2.17%) and moderate volatile matter (14.85–31.74%) reduced heating risks, while higher carbon content (46.27–69.48%) further mitigated the hazard. By integrating AI into coal mine safety protocols, this research offers a more efficient and predictive approach to managing thermal risks, reducing reliance on time-consuming laboratory testing.^45^

These case studies exemplify the transformative role of AI and ML in calorimetry and thermal systems, showcasing advancements in fire detection, wearable thermal management, self-powered safety systems, and predictive risk assessment. The integration of intelligent algorithms with real-time sensing and explainable AI fosters innovations that enhance safety, efficiency, and practical applications across various industries.

#### Comparative discussion of artificial intelligence/machine learning methods in calorimetry and thermal systems

Recent advancements in artificial intelligence (AI) and machine learning (ML) have catalyzed significant innovations in calorimetry and thermal management, particularly within renewable energy systems and fire safety applications. This section presents a comparative discussion of prominent AI/ML methods used in these domains, highlighting their practical applications, performance outcomes, and associated limitations based on recent literature and studies summarized in Table 2.

**Table 2:** Comparative summary of AI/ML techniques in calorimetry and thermal systems

AI/ML Method	Some Application Area	Key Outcome	Strength	Limitation	References
ANN/MLP/DNN/RNN	Heat exchangers, PCM, fire safety	High accuracy (R^2^∼0.99)	Flexible nonlinear modeling	Data-hungry, overfitting risk	Said et al.^8^; Ullah et al.^26^; Rehman et al.^31^; Naser^46^
CNN (e.g., U-Net, Swin-Unet)	Flame calorimetry, fire quantification	Low error (<20%)	Spatial feature extraction	High computational cost	Wang et al.^28^
ANFIS/Fuzzy Logic	TES, HVAC optimization	Balanced interpretability	Handles uncertainty	Complex to tune	He et al.^29^; Adelekan et al.^47^; Sharma et al.^50^
Random Forest, XGBoost, SVM, …	Nanofluids, coal mine heating, composites	Robust & reliable	Less overfitting	Limited transparency	Atofarati et al.^24^; Bifulco et al.^41^; Mishra et al.^45^; Adogbeji et al.^51^; Sharma et al.^52^
Reinforcement Learning (RL)	Heat sinks, adaptive system control	Adaptive optimization	Learning dynamic environments	High resource demand	Aldaghi et al.^34^
Symbolic Regression, XAI	Nanofluid modeling, thermophysical analysis	Interpretable models	Enhanced trust and insight	Still emerging, validation needed	Bhattad and Priyadarsan^35^; Kanti et al.^42^

*Artificial Neural Networks (ANN) and Variants (MLP, DNN, RNN):* ANNs remain the most widely applied models for predictive tasks across various thermal systems, including heat exchangers,^8^ phase change material (PCM) systems,^30^ and fire dynamics modeling.^46^ Their nonlinear learning capacity enables highly accurate predictions (R^2^ values approaching 0.99) and effective real-time thermal behavior modeling. However, their performance heavily depends on large volumes of high-quality data and requires careful regularization to avoid overfitting.

*Convolutional Neural Networks (CNN) and Image-Based Deep Learning*: CNNs are increasingly utilized in image-driven thermal diagnostics, such as flame calorimetry and fire monitoring.^28^ These models have demonstrated low prediction errors (<20%) in heat release rate estimation and are often integrated into digital twin systems for spatial feature recognition. Their primary advantage lies in spatial data extraction, but the trade-off is their high computational demand.

*Adaptive Neuro-Fuzzy Inference Systems (ANFIS) and Fuzzy Logic:* ANFIS models, which merge the learning capabilities of neural networks with the rule-based logic of fuzzy systems, have been successfully applied in thermal energy storage (TES) systems and HVAC optimization.^29^^,^^47^ These models offer a balance between interpretability and predictive performance, particularly under uncertainty. However, their performance can be hindered by gradient issues and the complexity of tuning fuzzy membership functions.

*Machine Learning Algorithms (Random Forest, Gradient Boosting, XGBoost):* These models are particularly effective in applications involving structured and heterogeneous data, such as predicting nanofluid thermophysical properties,^24^^,^^51^ coal mine thermal risk evaluation,^45^ and fire resistance of composite materials.^41^ Their strengths lie in robustness and reduced overfitting sensitivity. However, they are often criticized for limited transparency, which can hinder interpretability in critical engineering applications.

*Reinforcement Learning (RL) and Hybrid Models:* RL techniques are emerging as powerful tools for real-time, adaptive control of complex thermal systems, including the predictive optimization of hybrid heat sinks.^34^ These models dynamically adjust to changing thermal conditions through reward-based learning. While promising, RL methods typically require extensive computational resources and the careful design of reward functions, which can limit their scalability.

*Symbolic Regression and Explainable AI (XAI):* Recent research has emphasized the importance of model transparency, leading to a growing interest in symbolic regression and XAI techniques. These methods have been applied to hybrid nanofluid modeling^35^ and thermophysical property prediction using SHAP (SHapley Additive exPlanations) values.^42^ These models improve scientific interpretability and user trust, although they remain relatively immature in terms of widespread adoption and validation.

Each AI/ML technique offers distinct advantages and trade-offs in the context of thermal and calorimetric applications. While neural networks and CNNs excel in prediction and spatial analysis tasks, ensemble models provide robustness with minimal overfitting. ANFIS stands out in uncertain environments, whereas symbolic and explainable models offer transparency crucial for safety-critical domains. Reinforcement learning is particularly suited for real-time adaptive optimization, but is computationally demanding. Future research should emphasize a hybrid architecture that combines the strengths of multiple techniques and prioritize model validation in real-world renewable energy systems.

### Internet of Things for enhanced monitoring and automation

The IoT revolutionizes calorimetry and heat transfer by enabling real-time data acquisition, remote system monitoring, and dynamic process adjustments. IoT-integrated sensors continuously track key thermal parameters such as temperature, heat flux, and energy flow, optimizing system performance in renewable energy applications.

#### Internet of Things-enabled smart calorimetric and heat transfer systems

The incorporation of the Internet of Things (IoT) into calorimetry and heat transfer systems has revolutionized real-time monitoring, data acquisition, and energy optimization. IoT-enabled sensors enhance thermal performance by providing continuous, high-precision measurements of temperature gradients, heat flux, and energy flow, allowing for adaptive control of renewable energy systems. These sensors, when coupled with AI-driven analytics, improve decision-making in applications such as phase change materials (PCM), nanofluid-based cooling systems, and geothermal heat extraction.

**Figure 6:**
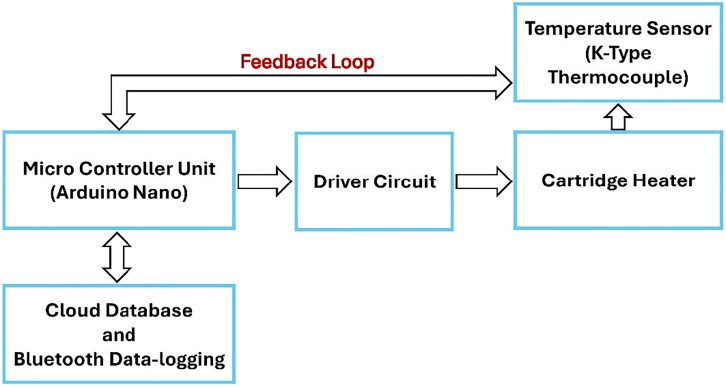
Diagrammatic depiction of the thermal management system Adapted from Kulkarni et al.^53^ published by IOP Science.

Kulkarni et al.^53^ developed an IOT portable microfluidic system for the synthesis of nanoparticles using Arduino-based microcontrollers with Bluetooth and cloud logging (Figure 6), the system showcases real-time data access and IOT-supported nanoparticle synthesis. Similarly, Xu et al.^54^ employed a system-on-chip (SoC) with CMOS MEMS technology to create a microcalorimetric flow sensor, achieving compactness, low cost, and bidirectional flow monitoring with high sensitivity. Meanwhile, Ding et al.^11^ innovated a self-powered thermoelectric fire warning system, leveraging IoT for ultrafast response and durability, essential for reliable fire safety applications. Liu et al.^55^ addressed energy efficiency in aluminum die-casting processes using IoT-enhanced incident control but acknowledged the necessity of broader process integration and data mining for continuous improvement. Table 3 presents a comparative analysis of various IoT-enabled calorimetric systems, showing their application types, accuracy, and power consumption, reinforcing the efficiency gains brought by IoT integration.

**Table 3:** IoT integration in calorimetry and heat transfer: Recent studies and insights

Author & Year	IoT Integration Method	Application Type	Purpose of Study	Implication of Study	Vital Findings	Additional Notes
Kulkarni et al.^53^ (2020)	Arduino + Bluetooth, cloud logging	Portable microfluidic system	MnO_2_ nanoparticle synthesis, testing	Real-time data access, geotagging, cloud storage; precise control up to 250°C	Precise MnO_2_ nanoparticle synthesis, electrochemical testing	Showcases IoT in portable automated thermal management
Gonzalez-Palacio et al.^56^ (2018)	SBC + IoT for real-time logging and prediction	IoT-enhanced autoclaves	Improve hospital sterilization steam quality	0.25% FS error (vs. 5.6% FS); enhanced predictive maintenance and near real-time monitoring	Affordable, accurate steam measurement	Highlights SBC and IoT cost-effectiveness in thermodynamic hospital applications
Xu et al.^9^ (2020)	Wireless dual-mode thermal flow sensor	Thermal flow sensors for HVAC	Extend flow measurement range	Range: 0–73 m/s; <2% error; wireless monitoring	Accurate IoT-based HVAC flow sensing	Future: integrate temp/humidity sensors for smart building energy monitoring
Xu et al.^54^ (2024)	Flexible sensing sheets for IoT	Flexible thermal sensors	Airflow and skin temp monitoring	∼50× accuracy boost; effective thermal barriers	Precise thermal sensing on curved surfaces, wearable devices	Future: healthcare monitoring, vacuum insulation, multiplexed detection
Dadhaneeya et al.^57^ (2024)	IoT-enhanced IR drying system	Food drying technology	Low-cost IR-assisted drying	Energy-saving; improved product quality; payback in 0.55 years	T-PET membrane proven superior for drying	Future: industrial scaling, material optimization
Prauzek et al.^10^ (2023)	IoT + energy harvesting + edge computing	Geothermal monitoring system	Monitor geothermal parameters	Reduced power via energy harvesting and edge computing	Identified efficient IoT designs for geothermal applications	Future: fault detection, advanced edge computing
Ding et al.^11^ (2024)	Bluetooth + ADC converter for wireless transmission	Thermoelectric fire warning system	Self-powered, remote fire warning	Ultrafast response (∼0.1s at 1 mV); durable for 180 days	Flame-retardant SWCNT/titanium carbide composite films used	Combines durability, fast response, and IoT-driven fire monitoring
Yaïci et al.^58^ (2022)	IoT control system with integrated sensors	Smart HVAC	Optimize heating/cooling in homes	Reduced energy waste; efficient energy use	Simulated comfort zones maintained	Future: real-world testing for energy savings validation
Yi et al.^59^ (2024)	IoT-integrated Android app + PMV model	Air conditioning	Thermal comfort optimization	Enhanced comfort, lower energy consumption	Simulated user-defined settings adjusted temp/fan speed	Further testing needed to validate real-world benefits
W. Liu et al.^55^ (2020)	IoT + optimization + incident control	Aluminum die casting	Improve energy efficiency	Tackled key energy barriers; optimal parameters improved efficiency	Cost savings (5–9%), stock reduction (3.6%), lower holding energy (2%)	Validated in a factory; future: data mining, broader process integration
Boehler et al.^60^ (2021)	IoT + thermal simulations	Autoclave sterilization	Automate cycle detection	Improved safety; valid sterilization detection	Reliable cycle counting: lifetime extended to 100 days	Hardware redesign needed for enhanced response and compliance
Xiang et al.^49^ (2022)	Triboelectric nanogenerator + CNN + LabVIEW	Thermo-regulating e-skin	Dynamic thermoregulation, handwriting recognition	98.13% handwriting recognition accuracy; IoT-driven thermoregulation	Real-time handwriting recognition; effective e-skin thermoregulation	Demonstrates universal IoT e-skin applications
Sharmila et al.^61^ (2020)	IoT + ThingSpeak for remote access	Transformer maintenance system	Monitor transformer oil performance	Automated critical oil property checks	Results guide oil replacement, purification, or retention	Future: automated *in situ* testing
Zhao et al.^62^ (2023)	PILGs for IoT fire systems	Fire protection	Self-powered, durable IoT fire protection	Ionic conductivity; rapid self-extinguishing	PILGs enable IoT-driven fire warnings and low-grade heat harvesting	Combines flame retardancy with IoT-based intelligent fire protection
Shafi et al.^63^ (2023)	IoT + ML (ANN)	Cotton storage SC detection	Detect/predict SC for fire prevention	ANN model: 99.8% accuracy; reduced fire hazards	IoT sensing ensures real-time SC prediction, protecting quality	Future: refining detection models, expanding IoT features
Sahu et al.^64^ (2024)	IoT multimode reflectance colorimeter	Gel crystallization monitoring	Monitor crystallization process	Real-time data; improved accuracy vs. commercial devices	Strong DSC correlation; tracked crystallization kinetics	Operates in testing/monitoring modes, applicable to food products such as butter

Another significant application of IoT in thermal energy management is in Heating, Ventilation, and Air Conditioning (HVAC) systems. Yaïci et al.^58^ developed an IoT-driven energy-efficient HVAC framework that dynamically adjusted airflow based on real-time thermal feedback. Their system reduced energy waste while maintaining optimal indoor climate conditions, where IoT-based dynamic thermal control is compared against conventional HVAC energy consumption patterns. The integration of IoT with big data analytics further optimized energy utilization, leading to a 15% reduction in operational costs and 21% and 30% improvement in energy consumed in heating and cooling conditions, respectively.

Despite these advancements, current IoT-enabled calorimetric and heat transfer systems face challenges related to data security, infrastructure scalability, and sensor durability in extreme thermal environments. Additionally, few studies have explored the integration of IoT with digital twins for predictive thermal simulations, leaving a gap in research on how IoT can enhance real-time feedback loops in advanced heat transfer applications. Future studies should focus on developing self-powered, low-energy IoT sensors with blockchain-based security frameworks to enhance data integrity and ensure robust deployment in large-scale renewable energy systems.

#### Case studies on Internet of Things in calorimetry and heat transfer

The integration of the Internet of Things (IoT) in calorimetry and heat transfer applications has significantly advanced monitoring, sensing, and control systems. Various studies have demonstrated the potential of IoT-driven technologies in different domains, including security, fluid flow sensing, fire safety, and wearable electronics.

##### Extraneous Sensors for Explosives Detection

**Figure 7:**
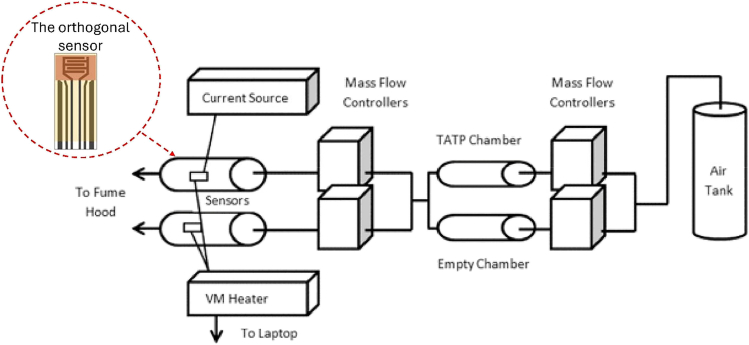
Schematic layout of the IOT-based explosive detector Adapted from Ricci et al.^65^ published by IEEE Xplore.

The development of an orthogonal sensor system designed for the trace detection of explosives was carried out by Ricci et al.,^65^ with the schematic layout shown on Figure 7. The platform employed a combination of thermodynamic and conductometric sensing methods to detect triacetone triperoxide (TATP) and 2,4-dinitrotoluene (2,4-DNT) in vapor form. The thermodynamic approach used thin-film microheaters with metal oxide catalysts on ultrathin alumina ceramic substrates, while the conductometric method measured changes in electrical resistivity as the catalyst interacted with explosive vapors. The study found that the sensor could detect TATP and 2,4-DNT at parts per billion (ppb) levels, demonstrating high sensitivity. The orthogonal approach improved system reliability by cross-verifying detection events, reducing false positives or negatives. The continuous, real-time monitoring capability aligns with IoT principles, facilitating enhanced security and environmental monitoring applications.^65^

##### IoT-Based Flow Calorimetry

A study by Xu et al.^9^ introduced a three-dimensional (3D) integrated micro calorimetric flow sensor utilizing Complementary Metal-Oxide-Semiconductor (CMOS) Micro-Electro-Mechanical Systems (MEMS) technology. The sensor featured a thermo-resistive micro calorimetric design with molybdenum (Mo) as the sensing material, housed within a sealed microchannel. A constant temperature control circuit ensured stable operating conditions, enhancing measurement accuracy. Results indicated high sensitivity to fluid flow changes, with a compact and efficient design enabled by 3D integration at the wafer level. The sensor’s compactness and accuracy make it particularly suited for IoT applications requiring real-time flow monitoring. This advancement has potential applications in biomedical devices, industrial process monitoring, and environmental sensing, where precise heat transfer measurements are critical.^9^

##### Ultrafast Intelligent Automated IoT Fire Alarm

**Figure 8:**
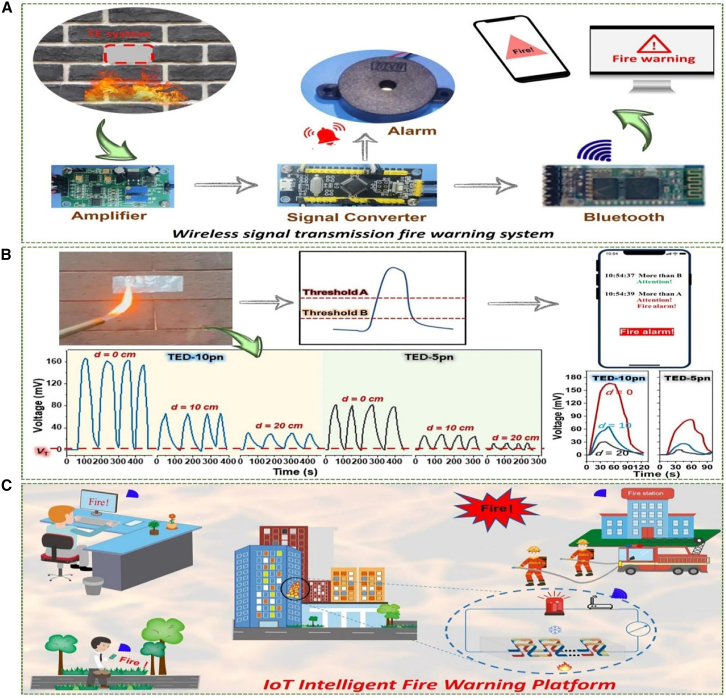
Thermoelectric-powered IoT system for fire warning applications (A) Schematic of the setup, (B) voltage response at different flame distances, and (C) MXene TE composites. Reproduced with Permission from Ding et al.^11^ under CC-BY License, published by Springer Nature.

Ding et al.^11^ developed an intelligent, self-powered IoT fire warning system using single-walled carbon nanotube/titanium carbide (SWCNT/Ti_3_C_2_) thermoelectric composite films. The system was designed for rapid fire detection, with composite films exhibiting high-temperature stability and flame retardancy. The thermoelectric devices (TEDs) were integrated with an amplifier, an analog-to-digital converter, and Bluetooth module for wireless communication as schematically presented in Figure 8.

Key findings included an ultrafast fire detection response time of approximately 0.1 s at a threshold voltage of 1 mV. The adjustable threshold voltage (ranging from 1 to 10 mV) allowed customization based on specific application needs. Additionally, the system demonstrated long-term stability over 50 repeated cycles and remained functional after 180 days of air exposure. This study contributes to the development of next-generation fire safety systems by leveraging IoT for real-time monitoring and self-powered operation. The flexible nature of the thermoelectric films enables easy integration into various environments, including textiles and building materials, thereby enhancing fire safety in industrial and residential settings.^11^

##### Smart Thermoregulating E-Skin

**Figure 9:**
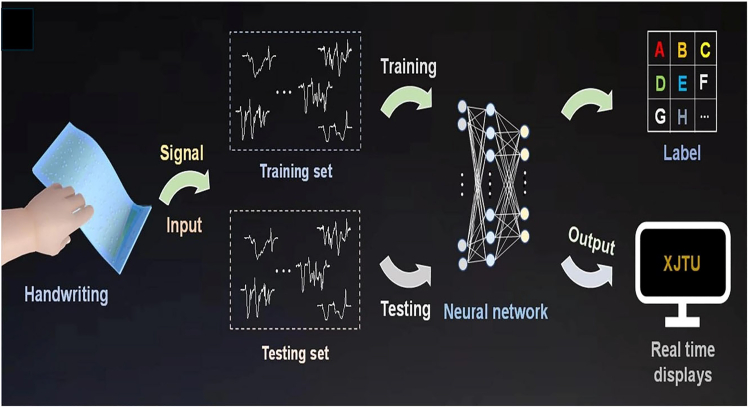
IoT-enabled deep learning-based real-time handwriting recognition system Reproduced with Permission from Xiang et al.^49^ under CC-BY License, published by Nature.

Xiang et al.^49^ developed an IoT-enabled electronic skin (e-skin) that integrates deep learning for handwriting recognition and dynamic thermoregulation. The e-skin was fabricated using flexible and stretchable materials, incorporating pressure sensors for handwriting detection and thermoelectric devices for temperature control. The schematics of this e-skin writing detector and display system is shown in Figure 9. A deep learning model was trained to recognize handwriting patterns, ensuring a personalized user experience. The study demonstrated that the e-skin could recognize handwriting in real-time while maintaining effective temperature regulation. The device showed high flexibility and durability, making it suitable for long-term wearable applications. The IoT connectivity enabled real-time data transmission, supporting applications in healthcare, personal authentication, and human-computer interaction.

This research underscores the potential of integrating deep learning, IoT, and thermal management in wearable technology, paving the way for future smart devices that offer enhanced interactivity, adaptability, and user comfort.^49^ These case studies illustrate how IoT is transforming calorimetry and heat transfer applications across multiple domains. From security and industrial monitoring to fire safety and wearable electronics, the integration of advanced sensors, deep learning, and real-time data connectivity enables precise, efficient, and responsive solutions. As IoT technology continues to evolve, further innovations in smart sensing, data analytics, and remote monitoring will enhance its applicability in various engineering and scientific fields.

### Big data analytics and cloud computing for scalable heat transfer optimization

The increasing complexity of renewable energy systems necessitates advanced data-driven approaches for optimizing heat transfer and energy management. Big data analytics and cloud computing have emerged as transformative tools in handling vast amounts of thermal data, enhancing predictive modeling accuracy, and ensuring efficient decision-making in calorimetric and heat transfer applications. The integration of AI, IoT, and cloud platforms enables real-time monitoring, predictive maintenance, and system optimization, significantly improving energy efficiency and reducing operational costs.

#### Enhanced data processing and real-time performance optimization

**Figure 10:**
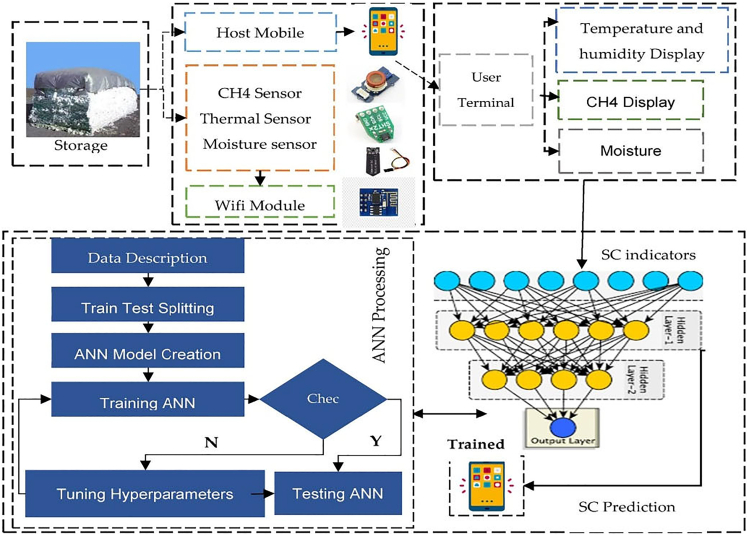
Integrated architecture for deep learning-based for fire prediction and prevention Reproduced with Permission from Shafi et al.^63^ under CC-BY License, published by MDPI.

Big data analytics accelerate real-time thermal performance evaluation by analyzing extensive datasets to identify trends, predict inefficiencies, and optimize energy flow. Shafi et al.^63^ demonstrated how artificial neural network (ANN) algorithms are applied to large-scale heat transfer datasets to accurately sense combustion of agricultural storage (Cotton) in real time, to prevent fire incidents as presented on Figure 10. The algorithm had an accuracy of 99.8% for the test data. Similarly, Sahu et al.^64^ applied IoT-driven big data analytics in crystallization monitoring systems, achieving precise heat transfer control and process optimization. These findings underscore the role of big data in automating complex thermal energy assessments, reducing dependency on manual calculations, and accelerating system responsiveness.

#### Cloud-based predictive maintenance and system optimization

Cloud computing has become indispensable for managing and storing the extensive data generated by IoT-enhanced calorimetric and thermal systems. The study by Kulkarni et al.^53^ demonstrates the effectiveness of cloud-based data logging in providing real-time access, geotagging, and efficient storage in portable thermal management systems. Similarly, Prauzek et al.^10^ highlights the integration of IoT sensors with cloud services for geothermal energy monitoring, emphasizing the role of cloud platforms in optimizing data collection and reducing power consumption. The work of Boehler et al.^60^ further explores cloud computing for thermal simulations in steam sterilization processes, ensuring enhanced system safety and reliability.

The scalability of cloud-based platforms allows for seamless handling of growing datasets, supports predictive maintenance, and ensures the availability of critical data across distributed systems. However, challenges such as ensuring data security, maintaining robust transmission speeds, and integrating edge computing technologies need to be addressed. Future advancements are likely to focus on developing more secure and efficient cloud solutions, leveraging AI-driven analytics, and incorporating edge computing to reduce latency and enhance real-time operations.

Despite its advantages, the implementation of big data analytics and cloud computing in heat transfer optimization faces challenges, including high computational demands, data privacy concerns, and interoperability issues between different cloud platforms. Current research primarily focuses on individual applications, with limited studies evaluating the combined impact of AI, big data, and cloud computing on scalable heat transfer optimization. Future research should develop energy-efficient cloud architectures that reduce computational overhead while maintaining predictive accuracy. Also, the need to investigate hybrid AI-cloud frameworks for real-time adaptive control in renewable heat transfer systems and address data security challenges by integrating blockchain-based encryption in cloud computing environments to protect sensitive energy data.

By integrating big data analytics with cloud computing, renewable energy applications can achieve greater scalability, predictive accuracy, and cost efficiency in heat transfer and calorimetric systems. However, addressing computational constraints, security risks, and system integration challenges is critical for maximizing the full potential of these technologies.

### Cyber-physical systems and digital twin technology

The advent of cyber-physical systems (CPS) and digital twin technology has introduced a transformative approach to the monitoring, control, and simulation of calorimetric and heat transfer systems. These advanced frameworks seamlessly integrate physical devices with computational models, enabling dynamic system analysis, predictive maintenance, and improved operational efficiency.

#### Integration of physical calorimetric devices with computational models

The integration of CPS into calorimetric systems has enhanced real-time interaction between physical devices and computational algorithms for enhanced monitoring and control. For instance, Xiang et al.^49^ developed a thermoregulating e-skin that combines triboelectric nanogenerators and real-time recognition using convolutional neural networks (CNNs). This system dynamically adapts to thermal conditions, offering universal thermoregulation and IoT-driven handwriting recognition with remarkable accuracy. Similarly, Xu et al.^9^ introduced a microcalorimetric flow sensor using system-on-chip (SoC) technology, achieving bidirectional gas flow measurement with high sensitivity and compact design. These studies highlight how CPS enhances dynamic system performance and optimizes energy usage in diverse applications.

#### Digital twins for virtual simulations and predictive maintenance in renewable energy systems

Digital twin technology has emerged as a powerful tool for simulating and predicting the behavior of renewable energy systems under varying conditions. For example, Gonzalez-Palacio et al.^56^ leveraged IoT-enhanced sterilization autoclaves combined with predictive models to improve real-time monitoring and maintenance. By incorporating these computational tools, the system reduced errors and enhanced reliability. Similarly, Prauzek et al.^10^ utilized IoT-integrated edge computing and cloud solutions to monitor geothermal energy installations, laying the foundation for digital twin applications in large-scale energy systems.

The adoption of digital twins allows for virtual simulations that replicate physical processes, enabling system optimization and early detection of faults. This approach enhances predictive maintenance strategies, minimizes downtime, and supports the development of resilient renewable energy infrastructure. However, challenges such as integrating diverse sensor networks, ensuring data accuracy, and developing scalable computational frameworks must be addressed to fully realize the potential of CPS and digital twins.

In conclusion, CPS and digital twin technology represent a paradigm shift in the management of calorimetric and heat transfer systems. Their ability to provide dynamic monitoring, predictive maintenance, and virtual simulations underscores their critical role in advancing the performance and sustainability of renewable energy systems.

### Blockchain for data security

Blockchain technology has gained significant attention for its potential to enhance data security and transparency in renewable energy systems. By leveraging distributed ledgers, blockchain ensures secure storage, traceability, and integrity of calorimetric and thermal data, addressing concerns related to data breaches and fostering trust in energy transactions.

#### Potential of blockchain technology in renewable energy transactions

Blockchain technology offers a robust framework for safeguarding calorimetric data and ensuring transparent and tamper-proof recording of energy-related activities. For example, in IoT-based systems such as those described by Ding et al.^11^ and Prauzek et al.,^10^ the integration of blockchain could add an additional layer of security to sensitive data such as geothermal system metrics or fire warning signals. These systems often handle real-time data, which could benefit from blockchain’s decentralized nature to prevent unauthorized access and guarantee authenticity. Additionally, blockchain’s immutability could enhance transparency in renewable energy transactions, such as recording energy generation, storage, and distribution.

#### Challenges and opportunities in implementing blockchain solutions for data integrity

Despite its promise, implementing blockchain in calorimetric and renewable energy systems faces several challenges. High computational requirements and energy consumption associated with blockchain protocols, especially for public blockchains, can limit their adoption in energy-sensitive systems. Integration with existing IoT networks and ensuring seamless interoperability with physical devices, as in Xu et al.^9^ and Xu et al.,^54^ requires sophisticated system architectures.

On the other hand, opportunities abound in developing tailored blockchain solutions for energy-efficient applications. For instance, lightweight blockchain protocols designed for IoT-enhanced calorimetry could optimize performance without compromising security. Furthermore, combining blockchain with smart contracts could automate renewable energy transactions and improve operational efficiency by reducing reliance on intermediaries.

In conclusion, blockchain technology holds immense potential to revolutionize data security and transparency in calorimetric and renewable energy systems. Addressing the associated challenges could unlock a future where secure, efficient, and transparent energy solutions are accessible and sustainable.

## Case studies of Fourth Industrial Revolution-Enhanced calorimetry and heat transfer in renewable energy

This section presents case studies illustrating how these technologies enhance efficiency, reliability, and scalability in solar, wind, geothermal, and bioenergy applications.

### Solar energy systems

Solar energy systems rely on efficient thermal management to maximize energy conversion and minimize losses as excess heat reduces these systems.^66^ AI, IoT, and calorimetry-driven solutions have been implemented to enhance photovoltaic (PV) performance, optimize thermal energy storage (TES), and improve overall system efficiency.

#### Artificial intelligence and Internet of Things for enhanced photovoltaic system performance

Advanced AI-driven algorithms and IoT sensors enable real-time efficiency tracking and predictive maintenance in solar PV systems.^67^ Yaïci et al.^58^ demonstrated the feasibility of IoT-based control systems in optimizing heating and cooling processes, which can be adapted for PV thermal management. Their system reduced energy waste and enhanced efficiency by dynamically adjusting operational parameters based on environmental conditions.

Xu et al.^54^ developed flexible IoT-enabled thermal sensors that monitor airflow and detect temperature anomalies in PV modules. By preventing overheating and improving energy output consistency, these sensors contribute to higher system reliability and lifespan.

#### Calorimetric analysis of thermal energy storage for solar applications

Calorimetry plays a critical role in evaluating TES systems by measuring the thermal properties and energy retention capabilities of storage materials. The development of an IoT-enhanced calorimetric system to assess nanofluid-based TES performance was carried out by Kulkarni et al.^53^ Their study revealed that precise real-time thermal monitoring led to improved energy storage efficiency and reduced losses in the solar-assisted heat exchangers considered. Gonzalez-Palacio et al.^56^ applied IoT-based data logging to optimize sterilization systems, demonstrating a framework that could be extended to solar TES. Their findings highlighted the importance of real-time calorimetric data acquisition in improving energy storage materials for sustainable solar applications. AI and IoT improve predictive maintenance and real-time monitoring in PV systems, while calorimetry enhances the evaluation and optimization of TES materials, leading to more efficient solar energy storage solutions.

### Wind energy systems

Wind turbine components are subject to variable thermal loads that can affect their efficiency and longevity. AI-powered predictive analytics and IoT-enabled condition monitoring systems have been instrumental in optimizing heat distribution and performance management.

#### Artificial intelligence-based predictive maintenance for thermal management in wind turbines

**Figure 11:**
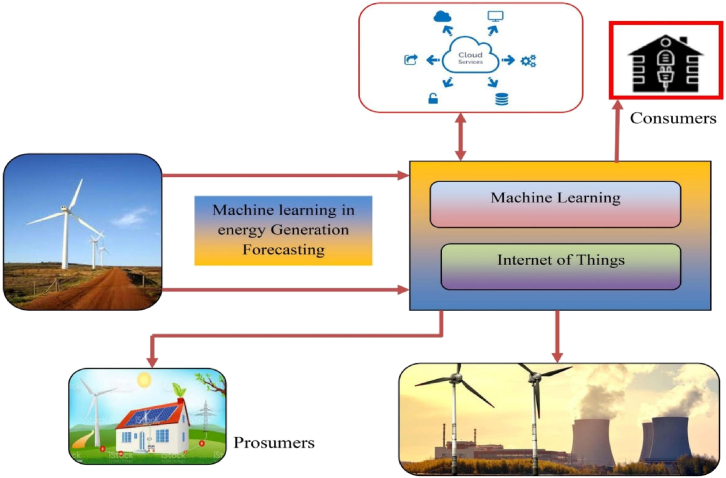
IOT enhanced wind-energy system Reproduced with permission from Kalpana et al.^68^ published by Taylor and Francis.

IoT technologies have been extensively utilized for monitoring and optimizing wind turbine operations, including the thermal performance of critical components. Kalpana et al.^68^ demonstrated the effectiveness of IoT in wind energy systems by improving wind speed forecasting and power generation accuracy as shown in Figure 11. These improvements directly impact thermal analysis, as precise energy prediction ensures stable thermal loads across components. IoT-enhanced systems enhance real-time data acquisition and analysis, facilitating the early detection of overheating or suboptimal thermal conditions in key components such as generators and gearboxes. This proactive approach minimizes downtime, reduces maintenance costs, and enhances overall system efficiency. Moreover, advancements in 3D printing and deep neural networks, as highlighted by Shin et al.,^69^ present cost-effective solutions for integrating IoT sensors into wind turbine designs. These sensors enable precise monitoring of thermal parameters and support adaptive adjustments for improved thermal performance in the wind turbine components.

Predictive analytics, powered by machine learning models, has proven invaluable for monitoring heat distribution and assessing the health of wind turbine components. Studies such as Hsu et al.^70^ and Leahy et al.^71^ illustrate the utility of statistical process control (SPC) and machine learning algorithms for fault diagnosis and predictive maintenance. These methods enable accurate predictions of component failures, including those related to thermal stress, by analyzing historical and real-time data. The integration of deep learning models, such as those described by Attallah et al.,^72^ further refines fault classification and severity analysis. For instance, ensemble models such as Inception and Xception achieve high accuracy in diagnosing thermal-related faults with minimal features and reduced computational overhead. Similarly, the work by Helbing and Ritter^73^ highlights the importance of supervisory control and data acquisition (SCADA) data in improving predictive maintenance, though challenges such as data resolution and class imbalance remain areas for future development.

### Geothermal energy systems

Geothermal energy relies on accurate reservoir characterization and optimized heat transfer processes to maximize efficiency. AI and calorimetric techniques have significantly improved the monitoring and management of geothermal systems.

#### Artificial intelligence/machine learning-enhanced geothermal reservoir characterization and heat transfer optimization

Moraga et al.^22^ introduced an AI-based framework integrating Remote Sensing (RS) and ML for geothermal site exploration. This methodology utilized indicators such as surface mineral markers, temperature anomalies, and fault structures to predict geothermal potential with remarkable accuracy (92%–95%). The proposed system demonstrated robustness by identifying hidden geothermal systems, as evidenced by a cross-site application with prediction accuracy between 72% and 76%. Such results underscore the utility of AI in mitigating geological and economic uncertainties associated with geothermal projects. Similarly, AlGaiar et al.^74^ emphasized the growing role of neural networks, support vector machines, and decision trees in geothermal exploration. These algorithms predict subsurface properties, such as rock permeability and fluid content, with improved precision, enabling the identification of optimal drilling locations. Despite challenges such as data quality and model transferability, their findings highlight how AI-driven insights reduce exploration risks and streamline project planning.

Geothermal-4IR technologies, as discussed by Muther et al.,^75^ further enhance reservoir management by integrating AI/ML into performance monitoring, maintenance evaluation, and reservoir storage optimization. These advancements facilitate real-time data analysis, enabling adaptive management strategies that optimize heat extraction while minimizing operational costs.

#### Calorimetric techniques for assessing geothermal heat transfer efficiency

Calorimetry provides precise measurements of geothermal fluid properties, ensuring accurate heat transfer analysis. Schröder et al.^76^ developed a flow calorimeter capable of measuring the specific heat capacity of geothermal water directly at power plant sites. The device’s design accommodates high temperatures (up to 170°C) and pressures (up to 3 MPa), ensuring compatibility with extreme geothermal environments. The experimental results exhibited deviations of less than 1% from reference data, confirming the system’s accuracy and reliability. This innovation addresses a significant knowledge gap by accounting for dissolved gases and minerals that influence heat capacity, thereby providing actionable insights for system design and optimization.

Integrating such calorimetric data with AI systems presents a promising avenue for improving geothermal heat extraction. For instance, AI-driven analysis can correlate calorimetric findings with operational parameters to predict long-term system performance. AlGaiar et al.^77^ suggested that combining calorimetric techniques with ML algorithms could enhance the understanding of heat transfer dynamics, leading to more efficient energy conversion processes. Additionally, integrating calorimetric systems with AI-enhanced predictive models can enable real-time monitoring and adaptive optimization of geothermal operations, paving the way for next-generation sustainable energy solutions. In summary, AI/ML improves geothermal site characterization and drilling accuracy, while calorimetry ensures precise heat transfer analysis for optimizing geothermal energy extraction.

### Bioenergy and biomass systems

Biomass and bioenergy technologies require real-time monitoring and optimization to maximize energy yields and ensure system sustainability. AI, IoT, and calorimetric analysis have significantly advanced biomass characterization and biofuel thermal stability evaluation.

#### Real-time calorimetry for biomass characterization and biofuel stability

Calorimetric techniques play a vital role in assessing biomass fuel quality and combustion efficiency. The study by Waqas et al.^78^ underscores the significance of high heating values (HHVs) in biomass characterization, where various biomass types, such as coconut leaves, date leaves, grass, date branches, and so forth, were examined. Meanwhile, cooked food waste demonstrated superior calorific values. These insights are critical for identifying optimal feedstocks for thermochemical and biochemical energy conversion technologies.

Moreover, the application of calorimetry extends to the evaluation of biofuel thermal stability, a factor essential for ensuring consistent energy yields and storage viability. Techniques such as bomb calorimetry have been effectively utilized to experimentally validate predictive models for biomass HHVs, as highlighted in Waqas et al.^78^ The integration of such methodologies allows for the comprehensive assessment of feedstock properties, enabling the development of tailored conversion processes to maximize energy efficiency and minimize resource wastage.

#### Internet of Things-based dynamic monitoring in bioenergy systems

IoT sensors enhance the efficiency and reliability of bioenergy systems by continuously monitoring feedstock moisture levels, combustion efficiency, and thermal stability. The integration of Internet of Things (IoT) sensors has revolutionized the monitoring and management of bioenergy systems by enabling real-time data acquisition and analysis. IoT-based platforms, as discussed by Setiawan et al.,^79^ provide a framework for evaluating the potential of biomass resources, particularly in regions such as Indonesia with substantial agricultural residues. By leveraging IoT technologies, stakeholders can perform dynamic simulations to identify the most sustainable utilization pathways for biomass, aligning with low-carbon economic goals.

IoT-enhanced sensors facilitate the continuous monitoring of critical parameters such as feedstock moisture content, calorific value, and conversion efficiency. These capabilities enhance process control and decision-making across the biomass-to-bioenergy supply chain (B2BSC). Mirkouei^80^ emphasized the role of cyber-physical systems in real-time sensing and advanced data analytics, which significantly improve the reliability and resilience of bioenergy operations. Furthermore, the combination of IoT and AI technologies supports predictive maintenance and optimization, ensuring uninterrupted operation and higher system efficiency. The deployment of IoT technologies also addresses challenges related to feedstock variability and supply chain disruptions. By integrating real-time data streams into adaptive control systems, bioenergy facilities can dynamically adjust operational parameters to accommodate changing conditions, thus enhancing overall system sustainability and productivity.

Hence, the adoption of real-time calorimetry and IoT-based monitoring systems represents a transformative step in the advancement of bioenergy and biomass systems. These innovations not only improve the efficiency and reliability of bioenergy production but also contribute to the global transition toward sustainable energy solutions. The synergy between technological advancements and interdisciplinary approaches will be pivotal in addressing the challenges of biomass utilization and realizing its full potential as a renewable energy source.

This section explores how AI, IoT, heat transfer, and calorimetry are revolutionizing renewable energy systems by enhancing efficiency, real-time monitoring, and predictive analytics. In solar energy, these technologies optimize PV thermal performance and thermal energy storage efficiency, while in wind energy, predictive maintenance improves reliability, and IoT enables real-time fault detection. Geothermal energy benefits from improved reservoir characterization and enhanced heat transfer efficiency through calorimetry, whereas bioenergy sees advancements in biomass selection and biofuel monitoring. These innovations collectively contribute to the development of intelligent, efficient, and sustainable renewable energy solutions. However, challenges such as data integration, infrastructure compatibility, and high implementation costs must be addressed to fully leverage the potential of 4IR technologies.

## Advances and challenges of Fourth Industrial Revolution-enhanced calorimetry and heat transfer in renewable energy

The integration of Fourth Industrial Revolution (4IR) technologies into calorimetry and heat transfer applications has led to significant advancements in efficiency, real-time monitoring, and predictive analytics. However, despite these innovations, several challenges remain that hinder widespread adoption. This section discusses key advancements, technical and economic challenges, and data security concerns associated with 4IR-driven calorimetry and heat transfer solutions.

### Key advancements

Over the past decade, the integration of AI, IoT, big data analytics, and digital twin technologies has significantly transformed calorimetry and heat transfer methodologies within renewable energy systems. These advancements have enhanced predictive accuracy, improved real-time monitoring, and increased the scalability of thermal energy management.^53^ One of the most notable improvements has been in predictive modeling, where AI-driven techniques, such as artificial neural networks (ANN) and regression algorithms, have outperformed traditional analytical methods in heat transfer calculations. For instance, Said et al.^8^ demonstrated how MLP-ANN models accurately predict solar-assisted heat exchanger efficiency, a substantial improvement over conventional empirical correlations. These AI models enable more precise thermal energy predictions, optimizing heat transfer processes and reducing energy losses.

The deployment of IoT-enabled calorimetric sensors has also revolutionized real-time system monitoring by providing continuous data acquisition, anomaly detection, and dynamic process adjustments. Xu et al.^54^^,^^81^ found that IoT-integrated heat flux sensors significantly improved the reliability of thermal management in renewable energy applications, reducing system inefficiencies and enhancing energy conservation. This capability allows for adaptive thermal control, where renewable energy systems can automatically adjust operational parameters based on environmental conditions, ensuring optimal performance with minimal energy waste.^73^

Beyond individual sensor applications, digital twins and cyber-physical systems (CPS) have emerged as powerful tools for real-time simulations and system optimization. Digital twin technology allows for the creation of virtual replicas of thermal systems, enabling advanced performance monitoring and predictive fault detection. Prauzek et al.^10^ demonstrated that digital twins could simulate adaptive heat transfer strategies for solar, wind, and geothermal energy systems, allowing operators to test different configurations before implementing changes in physical infrastructure. This approach enhances the scalability and flexibility of renewable energy applications, as digital simulations reduce reliance on costly experimental setups and minimize downtime in energy production systems.^69^

Another critical advancement lies in AI-enhanced predictive maintenance, which has significantly improved the operational efficiency of renewable energy infrastructure. In thermal energy storage (TES) systems and wind turbine cooling applications, AI-driven predictive maintenance strategies have reduced equipment failures and unplanned downtime. Leahy et al.^71^ found that deep learning models applied to wind turbine management resulted in a less than 40% downtime. By integrating predictive analytics with IoT-driven monitoring, renewable energy facilities can transition from reactive maintenance to proactive system optimization, enhancing both economic and environmental sustainability.

Despite these significant advancements, challenges remain in fully integrating 4IR technologies into large-scale renewable energy infrastructure. Many AI models still require high computational power, limiting their applicability in resource-constrained environments. Additionally, data security concerns surrounding IoT-enabled monitoring systems must be addressed to ensure safe and reliable system deployment. However, the application of AI, IoT, big data, and digital twin technologies has revolutionized heat transfer optimization in renewable energy systems. These advancements enable higher predictive accuracy, real-time monitoring, adaptive simulations, and proactive maintenance, driving greater efficiency and scalability. However, computational demands, data security, and system integration remain key challenges that require further research and innovation.

### Technical and economic challenges

Despite the significant advancements brought about by Fourth Industrial Revolution (4IR) technologies in calorimetry and heat transfer applications, several technical and economic challenges hinder their widespread adoption.

#### Technical challenges

One major technical challenge is the issue of data quality. The reliability of Artificial Intelligence (AI) and Machine Learning (ML) models heavily depends on a high-quality dataset.^29^^,^^70^ Incomplete or noisy data can lead to reduced prediction accuracy, resulting in suboptimal system performance. For instance, Ullah et al.^26^ highlighted that poor data quality adversely affects the performance of AI models in thermal systems. Another limitation is scalability, which poses a significant challenge according to Prauzek et al.^10^ Expanding Internet of Things (IoT)-based monitoring systems to large-scale renewable energy operations requires substantial infrastructure investments, making scalability a concern.

Integration complexity is another critical issue. Seamlessly integrating AI, IoT, and digital twins with existing energy infrastructure is challenging due to compatibility issues.^58^ Yaïci et al.^58^ emphasized the difficulties in integrating new technologies with legacy systems, which can hinder the adoption of 4IR technologies. Additionally, advanced AI/ML algorithms, such as deep learning, require significant computational resources, making real-time processing challenging for energy-constrained systems.^82^ Bhattad and Priyadarsan^35^ noted that the high computational demands of these algorithms limit their applicability in real-time scenarios.

#### Economic challenges

High implementation costs are a significant economic barrier. Deploying AI-driven thermal optimization systems and IoT-enabled calorimetric sensors requires substantial initial investments, which can be prohibitive, especially for small-scale renewable energy projects. Gonzalez-Palacio et al.^56^ highlighted that the high costs associated with adopting new technologies deter smaller enterprises from implementation. Uncertainty regarding the return on investment further complicates decision-making. The long-term economic benefits of AI and IoT-enhanced thermal energy systems are often uncertain, making it challenging for stakeholders to justify the investments. Boehler et al.^60^ discussed the difficulties in quantifying the economic returns of implementing these advanced technologies.

The lack of standardization in protocols for integrating AI/ML and IoT in renewable energy systems leads to inefficiencies and interoperability issues.^69^ Ding et al.^11^ also pointed out that the absence of standardized protocols hampers seamless integration and operation of these technologies. Moreover, IoT-enabled monitoring systems often require continuous power, which can offset the energy savings achieved through improved thermal efficiency. Xu et al.^9^ noted that the energy consumption of IoT devices can negate the benefits gained from enhanced efficiency, posing a paradox in energy management.

The high costs, integration complexity, and computational demands among other challenges of 4IR technologies, pose significant barriers to their widespread adoption in renewable energy applications. Addressing these challenges requires collaborative efforts from researchers, policymakers, and industry stakeholders.

### Data privacy and security concerns

The reliance on interconnected IoT and AI-driven calorimetry and thermal systems introduces critical data security and privacy risks, such as cybersecurity vulnerabilities,^71^ data breaches,^81^ weak encryption protocols,^29^ insider threats,^82^ regulatory compliance challenges,^70^ limited security awareness.^58^, high costs of security implementation^69^ and interoperability risks,^31^ among others. IoT-based calorimetric systems are susceptible to cyberattacks, which could compromise operational data integrity and lead to system failures. Shafi et al.^63^ highlighted that these vulnerabilities can result in significant disruptions to thermal energy systems. Unauthorized access to sensitive energy usage data poses significant risks, particularly in large-scale smart grid applications. Prauzek et al.^10^ emphasized that such breaches can lead to privacy violations and undermine user trust in renewable energy technologies.

Many IoT sensors operate with inadequate encryption standards, making them vulnerable to data interception and security breaches. Kalpana et al.^68^ discussed that the lack of robust encryption mechanisms in IoT devices can expose critical data to malicious actors. Adhering to evolving data protection regulations, such as the General Data Protection Regulation (GDPR), is complex and costly for global renewable energy deployments. Yaïci et al.^58^ noted that compliance with these regulations requires significant resources and can be challenging for organizations operating across different jurisdictions.

Many stakeholders lack the necessary expertise to implement robust cybersecurity frameworks for AI- and IoT-enabled thermal energy systems. Leahy et al.^71^ highlighted that this lack of awareness can lead to inadequate security measures, increasing the risk of cyber threats. The adoption of blockchain-based security solutions and advanced encryption protocols is necessary to enhance the cybersecurity of AI/IoT-enabled calorimetry and heat transfer systems.

In summary, notable advancements include the utilization of AI and IoT for thermal monitoring, leading to improved system efficiency, predictive maintenance, and scalability. However, several challenges impede widespread adoption. Technically, issues such as data quality, high computational demands, and integration complexities present significant hurdles. Economically, substantial implementation costs and a lack of standardization pose barriers, particularly for small-scale renewable energy projects. Moreover, IoT-enabled thermal systems face security concerns, necessitating stronger encryption, robust cybersecurity protocols, and adherence to regulatory compliance measures. Addressing these challenges requires future research focused on optimizing AI models for energy-efficient processing, enhancing the interoperability of IoT sensors, and developing standardized frameworks for secure data management in renewable energy systems.

### Sustainability benefits and policy challenges

The integration of 4IR technologies in calorimetry and heat transfer systems presents significant sustainability benefits, particularly in enhancing energy efficiency, reducing environmental impact, and optimizing resource utilization. However, despite these advantages, challenges related to environmental sustainability and policy barriers remain obstacles to large-scale adoption. Addressing these issues is crucial for ensuring the long-term viability of AI, IoT, and digital twin applications in renewable energy systems.

#### Sustainability benefits and environmental impact

The integration of 4IR technologies can optimize energy consumption and minimize waste for end users.^83^ For instance, AI-driven data analytics facilitate efficient energy management, reducing operational inefficiencies and associated emissions.^84^ IoT-enabled sensors provide real-time monitoring, allowing for proactive maintenance and reduced downtime, which contributes to lower energy consumption.^85^ Additionally, blockchain technology ensures the transparent tracking of energy transactions, promoting accountability and encouraging the use of renewable sources.^86^ The use of AI/ML and simulation for instead of some high risk experimentation involving nanomaterials among others are other benefits discussed by Atofarati et al.^87^ These applications collectively enhance environmental efficiency by reducing carbon footprints, reducing human risk to laboratory hazards, and promoting sustainable practices.

However, the deployment of 4IR technologies also presents environmental challenges.^88^ The manufacturing and disposal of electronic components can lead to increased electronic waste, and the energy demands of data centers supporting these technologies may offset some sustainability gains if not managed with renewable energy sources.^84^^,^^89^ The analysis of energy consumption of datacenters in the USA was carried out by Siddik et al.^90^ Their finding revealed that about 1.8% of the total energy consumed in the states was used by Datacenters. This excludes the fossil fuel consumption of some desert area data centers, which indirectly impacts the carbon footage and increases the global greenhouse gas emission.^91^^,^^92^ Moreover, large-scale renewable energy projects, such as wind and solar farms, can disrupt local ecosystems if not properly planned, leading to habitat fragmentation and biodiversity loss.^93^^,^^94^^,^^95^

#### Policy barriers to implementation

Despite the potential sustainability benefits, several policies and regulatory barriers hinder the large-scale implementation of 4IR-driven calorimetric and heat transfer systems.^96^^,^^97^ A lack of clear regulatory frameworks for integrating AI, IoT, and blockchain technologies into energy infrastructure creates uncertainty for investors and industry stakeholders.^98^ For example, data privacy laws^99^^,^^100^ and cybersecurity^101^^,^^102^ regulations remain fragmented, making it difficult to implement IoT-enabled thermal monitoring systems in compliance with existing standards. Countries with strict General Data Protection Regulation (GDPR) requirements impose additional constraints on cloud-based and AI-driven energy analytics, limiting the cross-border deployment of smart thermal management solutions.^103^^,^^104^

Additionally, the absence of standardized protocols for AI and IoT interoperability in calorimetric applications leads to fragmentation in technology deployment, where different industries and regions adopt incompatible solutions. Without internationally recognized technical standards, the widespread adoption of 4IR technologies in renewable energy remains slow and inconsistent. Furthermore, financial and policy incentives for AI-driven sustainability efforts are still limited, with most government subsidies focusing on traditional renewable energy infrastructure rather than digital optimization technologies as emphasized in the works of Danish et al.^105^^,^^106^

While 4IR technologies offer transformative sustainability benefits, including enhanced energy efficiency, reduced environmental impact, and improved material utilization, several policies and regulatory challenges continue to hinder their adoption. Addressing data security concerns, establishing standardized AI and IoT protocols, and introducing supportive government policies will be essential to accelerating the implementation of smart calorimetry and heat transfer solutions in renewable energy applications. Future research and policy initiatives should focus on developing sustainable thermal materials, strengthening regulatory frameworks, and promoting financial incentives to encourage wider adoption.

## Emerging trends and future research directions

The integration of Fourth Industrial Revolution (4IR) technologies into calorimetry and heat transfer applications continues to evolve, offering new possibilities for enhancing the efficiency and scalability of renewable energy systems. This section highlights emerging trends and outlines key research priorities for advancing 4IR-enabled calorimetry and heat transfer solutions.

### Emerging trends in Fourth Industrial Revolution technologies for calorimetry and heat transfer

Recent advancements in artificial intelligence (AI), the Internet of Things (IoT), big data analytics, and material science are revolutionizing calorimetry and heat transfer applications in renewable energy. These technologies enable greater predictive accuracy, real-time monitoring, enhanced energy efficiency, and improved system scalability. Current trends indicate a shift toward AI-driven predictive modeling, smart IoT-based thermal management, advanced sensor integration, and the broader application of 4IR technologies across renewable energy systems. As the demand for efficient thermal energy solutions grows, these emerging trends play a crucial role in optimizing cooling systems, phase change materials (PCM), hybrid nanofluids, and large-scale renewable energy infrastructure.

#### Artificial intelligence-driven predictive modeling for heat transfer optimization

AI and ML continue to refine predictive models for thermal energy applications, improving the design and performance of heat transfer systems. Artificial Neural Networks (ANN) and regression-based AI models have demonstrated significant improvements in predicting thermophysical properties and optimizing nanofluids for enhanced cooling efficiency.^26^ These models outperform traditional empirical correlations, reducing prediction errors and enabling precise control over fluid behavior in heat exchangers. Additionally, Recurrent Neural Networks (RNNs) and ML-driven models have facilitated the real-time monitoring and optimization of PCM thermal performance, improving their integration into thermal energy storage (TES) systems.^30^ Furthermore, advanced ML algorithms such as XGBoost and Symbolic Regression models presented by Bhattad & Priyadarsan^35^ and other researchers^38^^,^^107^ have been successfully applied to predict the thermal conductivity and specific heat capacity of hybrid nanofluids, aiding in the development of next-generation thermal management solutions. These AI-driven innovations provide more efficient, cost-effective, and scalable solutions for managing heat transfer in renewable energy applications.

#### Internet of Things for smart thermal management systems

The rapid adoption of IoT-enabled monitoring systems is significantly enhancing real-time heat transfer management across various renewable energy applications. In smart heating, ventilation, and air conditioning (HVAC) systems, IoT-driven climate control solutions dynamically adjust HVAC performance, reducing energy consumption while maintaining optimal indoor thermal conditions.^58^ Similarly, the development of self-powered IoT sensors, including triboelectric nanogenerators (TENGs) and thermoelectric materials, is providing more energy-efficient solutions for continuous thermal monitoring without external power sources.^10^^,^^53^ This advancement addresses one of the major limitations of conventional IoT sensors, which rely on constant energy inputs. Furthermore, IoT-integrated flow calorimeters are improving the accuracy of thermal property assessments in solar and geothermal energy applications, enhancing the efficiency of heat extraction and storage processes as seen in the work of Schröder et al.^76^ The ability of IoT devices to collect, analyze, and respond to thermal data in real time is reshaping heat transfer optimization, ensuring more adaptive and self-regulating thermal systems.

#### Advanced thermal sensing and data analytics

The integration of advanced sensor technologies and big data analytics is further enhancing the precision and responsiveness of calorimetric and heat transfer systems. The development of IoT-enabled thermoelectric sensors for fire warning systems has significantly improved fire detection speeds, reducing response times to as low as 0.1 s, thereby increasing safety in thermal energy applications.^11^ Similarly, AI-integrated flexible thermal sensors are improving airflow monitoring and surface temperature assessments, making them highly valuable for smart building applications and wearable heat transfer devices.^54^ Beyond sensing technologies, the exploration of blockchain-based security frameworks is becoming increasingly relevant in IoT-enabled thermal systems, ensuring data integrity and cybersecurity as recommended in the study by Prauzek et al.^10^ These advancements address critical challenges in real-time thermal management, providing faster, more accurate, and more secure heat transfer monitoring solutions.

#### Integration of Fourth Industrial Revolution technologies in renewable energy systems

The application of 4IR technologies in renewable energy continues to optimize thermal management in various sectors. In solar-assisted heat transfer systems, AI-driven ANN models are being leveraged to optimize heat exchangers, improve energy efficiency, and reduce greenhouse gas emissions.^8^ Similarly, in wind energy systems, deep learning models and 3D-printed IoT sensors are enhancing heat dissipation in wind turbines, minimizing energy losses caused by thermal fluctuations.^68^ The bioenergy sector is also experiencing efficiency gains, as real-time calorimetry and IoT-based monitoring systems enhance biomass-to-biofuel conversion, leading to higher energy output and reduced process inefficiencies.^78^^,^^79^ The continuous evolution of 4IR technologies in these applications is bridging the gap between digital transformation and practical energy solutions, allowing for more adaptive, scalable, and efficient thermal energy management.

In summary, AI, IoT, and advanced sensing technologies are driving transformative changes in calorimetry and heat transfer systems, enabling more efficient, intelligent, and sustainable thermal energy solutions. AI-driven predictive models are optimizing heat transfer through advanced computational algorithms, while IoT-enabled real-time monitoring ensures better energy management across renewable energy applications. Big data analytics and next-generation thermal sensors are enhancing the accuracy of thermal property assessments, leading to improved safety, efficiency, and performance. However, while these emerging trends offer significant improvements in renewable energy heat transfer optimization, further research is needed to improve integration, scalability, and security to ensure seamless deployment across industries.

### Recommendations for future research

To overcome existing challenges and maximize the potential of 4IR technologies in calorimetry and heat transfer, future research must address key areas such as hybrid AI-physics modeling, real-world validation, economic feasibility, and standardization. These research directions will help bridge the gap between advanced digital technologies and their large-scale implementation in renewable energy systems, ensuring scalability, efficiency, and long-term sustainability.

One crucial area for advancement is the development of hybrid AI-physics models that integrate machine learning algorithms with fundamental heat transfer equations. Current AI-driven models rely heavily on large-scale datasets, making them prone to inaccuracies when data are limited or noisy.^26^ By incorporating physics-based principles, AI models can achieve greater reliability, better generalization across different thermal systems, and reduce dependence on extensive training datasets. This hybrid approach will enhance the predictive accuracy of AI models in optimizing heat transfer processes and provide a more robust framework for AI-driven thermal management.^30^

Beyond model development, long-term field studies and real-world implementation are essential to validate the performance of AI- and IoT-enhanced calorimetric systems under actual operational conditions. While laboratory experiments and simulations offer valuable insights, scalability and adaptability to diverse environmental conditions remain a challenge. Future research should evaluate AI-powered predictive maintenance strategies in industrial settings, particularly in large-scale renewable energy plants, to assess their effectiveness in reducing downtime and optimizing system efficiency.^8^ Additionally, research should focus on IoT-enabled smart grid integration, investigating how calorimetric sensors can improve real-time energy distribution and grid stability.^58^ These studies will provide empirical data to support widespread adoption and regulatory approvals of AI- and IoT-driven heat transfer technologies.

Economic and environmental impact assessments are also critical for accelerating the adoption of 4IR technologies in heat transfer applications. To ensure financial viability, future studies should quantify cost savings from predictive maintenance, analyzing how AI-driven heat transfer optimization reduces energy waste and lowers operational expenses.^60^ Furthermore, sustainability assessments should measure carbon footprint reductions achieved through improved calorimetric efficiency in renewable energy applications.^79^ By demonstrating the economic and environmental benefits of 4IR-enhanced thermal systems, policymakers and industry stakeholders will be more inclined to invest in and implement these advanced technologies on a larger scale.

A significant barrier to adoption is the lack of standardization in AI- and IoT-driven thermal monitoring systems, which creates inefficiencies and hinders interoperability across different renewable energy infrastructures. Future research should prioritize the development of open-source AI models for heat transfer applications, ensuring transparency, accessibility, and cross-industry collaboration.^20^ Additionally, the creation of unified IoT communication protocols is essential for seamless integration between IoT-enabled calorimetry systems and existing renewable energy networks (Prauzek et al.^10^). Standardization efforts will enhance data consistency, improve device compatibility, and facilitate the large-scale deployment of smart calorimetric solutions.

To ensure the sustainable adoption of 4IR technologies in calorimetry and heat transfer applications, future research and policy initiatives must address both environmental sustainability and regulatory challenges. While AI, IoT, and blockchain enhance energy efficiency and resource optimization, their long-term environmental impact including the energy consumption of data centers, electronic waste from IoT devices, and the sustainability of advanced materials requires further investigation.^92^ Research should focus on developing eco-friendly, recyclable thermal materials and energy-efficient computational models to mitigate these concerns. Additionally, policy and regulatory barriers remain a significant obstacle to large-scale implementation. The lack of clear policies on AI and IoT integration, data privacy regulations, and standardization frameworks creates uncertainty for industry adoption. Governments and regulatory bodies must work toward harmonized standards for AI-driven heat transfer systems, financial incentives for sustainable technology adoption, and policies that ensure cybersecurity and data integrity. Without addressing these sustainability and policy barriers, the full potential of 4IR technologies in renewable energy systems will remain unrealized.

Future research should focus on advancing hybrid AI-physics models (Ullah et al.^26^; Anooj et al.^30^) to improve predictive accuracy and computational efficiency, conducting long-term real-world validation (Said et al.^8^; Yaïci et al.^58^) to assess practical implementation, performing economic and environmental assessments (Boehler et al.^60^; Setiawan et al.^79^) to quantify financial viability and sustainability, and establishing standardized protocols (Ding et al.^11^; Prauzek et al.^10^) to enhance interoperability and scalability. Addressing these key areas will ensure that 4IR technologies in calorimetry and heat transfer become more reliable, cost-effective, and widely adopted, driving the transition toward sustainable and intelligent thermal energy solutions.

This section explores the emerging trends and future research priorities in 4IR-driven calorimetry and heat transfer applications, emphasizing the transformative role of AI, IoT, and blockchain in optimizing thermal energy monitoring across solar, wind, geothermal, and bioenergy systems. These technologies are reshaping the way renewable energy systems manage heat transfer by enabling real-time monitoring, predictive maintenance, and improved energy efficiency. However, despite these advancements, several research gaps remain that must be addressed to maximize the potential of 4IR technologies in practical applications.

Future research should prioritize hybrid AI-physics modeling to enhance predictive accuracy and computational efficiency, ensuring that machine learning models integrate fundamental heat transfer equations for improved reliability. Additionally, long-term field validation studies are necessary to assess the real-world performance of AI and IoT-enabled thermal systems, particularly in large-scale renewable energy infrastructure. Economic and environmental impact assessments should also be conducted to quantify cost savings, sustainability metrics, and carbon footprint reductions associated with AI-driven heat transfer optimization. Moreover, the standardization of AI, IoT, and blockchain protocols is critical for ensuring seamless interoperability and facilitating the large-scale deployment of 4IR-enhanced calorimetry solutions.

By addressing these research challenges, the renewable energy sector can fully harness 4IR technologies to optimize heat transfer processes, improve system efficiency, and accelerate global sustainability and policy efforts. Continued innovation in this field will be essential for driving technological advancements, reducing operational costs, and ensuring a more resilient and energy-efficient future.

## Conclusion

The application of Industry 4.0 technologies, including artificial intelligence (AI), machine learning (ML), the Internet of Things (IoT), big data analytics, digital twins, and blockchain, is accelerating innovation in calorimetry and heat transfer for renewable energy systems. These technologies have enhanced predictive modeling accuracy, enabled real-time thermal diagnostics, and improved energy system reliability across solar, wind, geothermal, and bioenergy platforms.

This review has synthesized evidence from over 100 recent studies, highlighting the technical and operational benefits of 4IR tools, such as AI-driven heat transfer optimization, IoT-enabled condition monitoring, and blockchain-secured data handling. However, the practical deployment of these technologies remains constrained by high computational requirements, interoperability limitations, cybersecurity vulnerabilities, and inconsistent regulatory environments.

To advance the field, future research should focus on integrating data-driven AI models with physics-based thermal frameworks to improve robustness and generalizability. Emphasis should be placed on developing explainable AI architectures, validating performance through long-term field studies, and optimizing algorithms for low-power, resource-constrained settings typical in remote renewable energy applications. From a policy perspective, establishing region-specific standards for data security, interoperability, and safety compliance is essential to facilitate large-scale adoption.

Furthermore, sustainability considerations, such as the life cycle impacts of IoT devices, energy consumption of data centers, and the use of advanced thermal materials, must guide both research and deployment strategies. Addressing these environmental and systemic challenges will be critical to ensuring that 4IR-enabled calorimetry and heat transfer systems contribute meaningfully to decarbonization and global energy transition goals.

## Author contributions

Dr. Emmanuel O. Atofarati: involved in conducting literature search, analyze findings, and preparing the initial draft of the article. While Prof. Christopher C. Enweremadu: refining the methodology, reviewing, and revising the article.

## Declaration of interests

The authors confirm that there are no financial conflicts or personal relationships that could affect the integrity of the work presented in this article.
